# Carbon and Iron Uptake by Phytoplankton in the Amundsen Sea, Antarctica

**DOI:** 10.3390/biology11121760

**Published:** 2022-12-04

**Authors:** Bo Wang, Lingfang Fan, Minfang Zheng, Yusheng Qiu, Min Chen

**Affiliations:** 1 College of Ocean and Earth Sciences, Xiamen University, Xiamen 361102, China; 2 College of Safety and Environmental Engineering, Shandong University of Science and Technology, Qingdao 266590, China

**Keywords:** Fe uptake, carbon fixation, sea ice meltwater, meteoric water, Amundsen Sea

## Abstract

**Simple Summary:**

In the Amundsen Sea in late summer, sea ice meltwater has a more pronounced effect on the CFR and FeUR than meteoric water. Meteoric water, however, promotes the growth of larger phytoplankton that are susceptible to Fe deficiencies. Sea ice formation inhibits carbon fixation, resulting in a higher intracellular Fe/C ratio.

**Abstract:**

Freshwater components in the Southern Ocean, whether sea ice meltwater or meteoric water, influence the growth of phytoplankton by affecting water stability and supplying dissolved iron (DFe). In addition, melting sea ice stimulates phytoplankton blooms by providing ice algae. In this study, sea ice meltwater and meteoric water in the Amundsen Sea (AS) were differentiated by their stable oxygen isotopic compositions (δ^18^O), while the phytoplankton carbon fixation rate (CFR) and iron uptake rate (FeUR) values were determined using the ^14^C and ^55^Fe tracer assays, respectively. Our results showed that FeUR exhibits a significant positive response only to sea ice meltwater, suggesting that DFe and algae provided by sea ice melting may be the main cause. In addition, the CFR had a slightly positive response to the freshwater input and a stronger correlation with the phytoplankton biomass, suggesting that the freshwater input may have enhanced the CFR through the algae released from sea ice melting. The FeUR normalized to the phytoplankton biomass was significantly positively correlated with the mixed layer depth, suggesting that water stability regulates the phytoplankton growth and the resulting Fe demand. A higher Fe demand per unit of carbon fixation during sea ice formation leads to a higher Fe/C ratio in phytoplankton. Although no significant correlations were observed between the FeUR, CFR, and meteoric water, meteoric water may have an effect on larger phytoplankton sensitive to Fe deficiencies. The results of culture experiments with DFe addition showed that the added Fe significantly enhanced the Fe uptake, carbon fixation, and Fe/C ratio of the cells, especially for micro-phytoplankton. The more pronounced response of micro-phytoplankton means that the meteoric water input may affect the efficiency of carbon export. Our study provides the first measurements of phytoplankton Fe quotas in the AS in austral late summer and early autumn, providing insights into how meteoric water and sea ice meltwater affect seasonal changes in Antarctic ecosystems.

## 1. Introduction

The Southern Ocean (SO) has an impact on climate change by absorbing atmospheric CO_2_ through the primary production of phytoplankton. The primary production in the SO increases rapidly in the austral spring (late October or early November), driven by melting sea ice, increased solar radiation, and water stratification [1,2]. As major nutrients such as dissolved inorganic nitrogen (DIN) and dissolved inorganic phosphorus (DIP) are abundant, the intensity and spatiotemporal variation of the primary production in the SO is thought to be mainly regulated by dissolved Fe (DFe) and light [2,3,4,5,6,7,8]. Numerous studies have shown that the DFe concentration plays a key role in the primary productivity, while the light intensity modulates the phytoplankton community’s structure [2,9,10,11]. Diatoms and Antarctic *Phaeocystis* are often the dominant species in phytoplankton communities in the SO [9]. The changes in phytoplankton communities affect biological pump and carbon exports in the SO due to the different nutrient utilization and support rates for different zooplanktons [2]. In the context of climate warming, changes in ice–sea–air interactions will have an impact on the SO ecosystem, thereby affecting the absorption of atmospheric CO_2_ [6,7,10,12,13].

The polynyas are some of the unique areas in polar oceans that are much more productive than the open ocean [11,14]. Around the Amundsen Sea (AS), several polynyas formed by seasonal changes of sea ice appear every year, of which the Amundsen Polynya (AP) and the Pine Island Polynya (PIP) are the two larger and more productive ones. Between 2008 and 2009, the ice-free days for the AP and PIP reached 145 d, of which the maximum open water areas in early February reached 31,000 km^2^ and 197,000 km^2^, respectively. The primary productivity in these two polynyas was as high as 3 g C m^−2^ d^−1^, about 10 times the average for the entire SO [9]. The volume of glaciers around the AS is about 7 × 10^5^ km^3^, which is about one-third of the West Antarctic ice sheet. In recent decades, global warming has increased the temperature of the circumpolar deep water (CDW). These warmer waters rise up the Amundsen Shelf and interact with the bases of the Pine Glacier and the Getz and Dotson ice shelves, causing the glaciers and ice shelves to melt rapidly. It has been estimated that glaciers around the AS have been lost at an average rate of 51 ± 9 km^3^/a over the past few decades, and the sea ice coverage has decreased by about 20% since 1973 [15,16].

The phytoplankton biomass rates in the AS range from <1 μgChl *α* L^−1^ to ~ 40 μgChl *α* L^−1^, with the highest values tending to occur in steadily stratified waters. The DFe concentrations in the AS varied from 0.042 nM to 1.31 nM, with the lowest values occurring at sites with the highest Chl *α* content and the highest values near the Pine Island Glacier [11]. In the PIP, the subsurface minima of the DFe appeared at the depths (20–25 m) where the Chl *α* maxima were located, indicating that the distribution of the DFe was significantly affected by the phytoplankton uptake. The phytoplankton blooms in the PIP and AP in the summer of 2009 persisted for more than 70 days, and their available Fe was thought to be related to the meltwater from the sea ice and ice shelves. The intrusion of warmer modified circumpolar deep water (MCDW) exacerbates the melting of the glaciers and ice shelves, replenishing the polynyas with DFe and promoting algal blooms [11]. Furthermore, a positive correlation between the temperature and primary productivity was observed in the AS, proving that MCDW is one of the sources of DFe in the polynyas. 

The main purpose of this study was to explore the effects of sea ice meltwater and meteoric water on the uptake of dissolved inorganic carbon (DIC) and DFe by phytoplankton, thereby revealing the role of freshwater in regulating phytoplankton growth in the AS. To this end, we quantitatively differentiated the contributions of sea ice meltwater and meteoric water using the oxygen isotopic composition (δ^18^O) in seawater, and measured the carbon fixation rate (CFR) and Fe uptake rate by phytoplankton (FeUR) using culture experiments with the radionuclides ^14^C and ^55^Fe. By analyzing the relationships between freshwater components and CFR and FeUR, we try to find out how these freshwater components affect the DIC and Fe absorption by the phytoplankton and which freshwater component plays a more important role in the growth of phytoplankton in the AS.

## 2. Materials and Methods

### 2.1. Study Area

**Figure 1:**
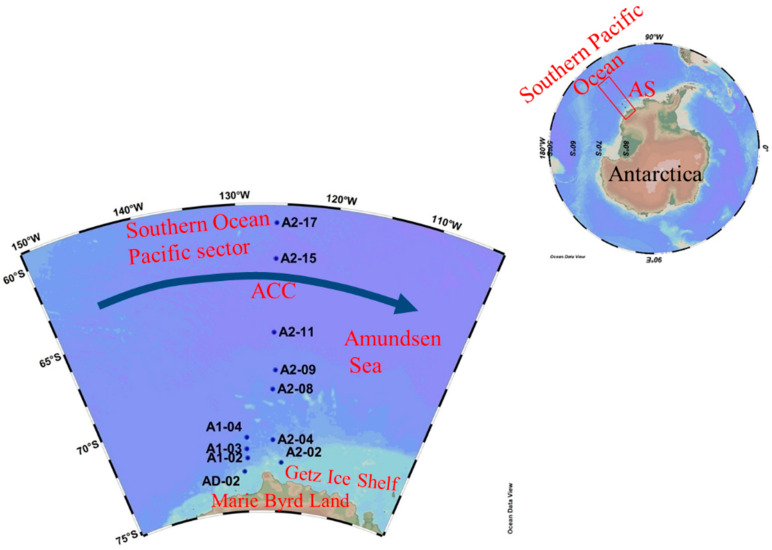
The sampling locations in the Amundsen Sea, Antarctica. The eastward-flowing Antarctic Circumpolar Current (ACC) is shown.

Our study area was the AS outside the Getz Ice Shelf, with the latitude and longitude values ranging from 60.00° S to 73.18° S and from 125.98° W to 130.38° W, respectively. The eastward-flowing Antarctic Circumpolar Current (ACC) divides our study area into the open ocean and continental shelf (Figure 1). The water mass in the open ocean is mainly CDW, while the water masses affecting the continental shelf include rising MCDW, winter water (WW), and Antarctic surface water (AASW) [17]. The DFe concentration in the CDW is lower (~0.3 nmol L^−1^), while the high temperature MCDW contains a higher DFe concentration (0.37 nmol L^−1^) and other inorganic nutrients due to the influence of meteoric meltwater [18]. The higher concentrations of major inorganic nutrients in the continental shelf in the AS (DIN: 26.1 ± 6.0 μmol L^−1^; DIP: 2.0 ± 0.4 μmol L^−1^; DSi: 77.0 ± 11.0 μmol L^−1^) are not thought to limit phytoplankton growth [19]. Previous studies have shown that the dominant species of phytoplankton in spring blooms in the AS is Antarctic *Phaeocystis*, while diatoms dominate in non-bloom areas [20]. Therefore, differences in water masses and the resulting nutrients may be some of the important factors affecting the growth and community structure of phytoplankton in our study area.

### 2.2. Sampling

Seawater samples were collected from March 3 to 15, 2018, along two meridional sections (A1 and A2) covering the ice margin zone, continental shelf, and open ocean. The sampling sites extend from the open ocean to the continental shelf, spanning the influence of the ACC (Figure 1). Since the sampling was carried out in late summer and early autumn, the temperatures were mostly below 0 °C and the sea ice coverage near the coast was high. The southernmost stations in the two sections are AD-02 (73.20° S) and A2-02 (72.74° S), respectively, and their water depths are both less than 500 m. Some lotus leaf ice had been formed at the time of sampling at these two stations, with a sea ice density range of 40–50%. Stations 02, 03, and 04 in section A1 and station 04 in section A2 are located on the outer edge of the continental shelf, with a water depth of about 3000 m. Station 08 in section A2 and the stations to the north are in open ocean with water depths ranging from 3750 m to 4800 m (Figure 1). 

Seawater was collected using the CTD rosette collector on board the R/V *Xuelong*. The hydrochemical parameters, including the temperature (T), conductivity, pressure, chlorophyll fluorescence intensity, and dissolved oxygen (DO), were measured using the CTD and other sensors [21]. The salinity was calculated from measurements of conductivity, temperature, and pressure (psu, 0–70, ±0.0003). 

The samples for measurements of the carbon fixation rate and Fe uptake rate were taken from the surface layer (~5 m depth) and at the depth of the Chl *α* maximum (CMD, determined from the down-cast fluorescence profile). A total of 44 stations and layers were measured in this study. 

All polycarbonate flasks, carboys, and Teflon wares (Nalgene^TM^) used in our experiments were pre-cleaned before use. The cleaning procedure mainly consisted of soaking in 1 M HCl (trace metal grade) for >24 h and washing in Milli-Q water (18.2 MΩ) at least 6 times before storage in a clean bench.

### 2.3. Determination of Major Nutrients

The nutrient samples were collected from the surface (~5 m depth) and the CMD depth. The nutrient pretreatment was described in our previous study [21]. The nitrate (NO_3_^−^), nitrite (NO_2_^−^), and ammonium (NH_4_^+^) were measured using the methods for cadmium–copper reduction, diazonium-azo, and indophenol blue, respectively [22]. The phosphate (PO_4_^3−^) and silicate (SiO_3_^2−^) were determined using the methods for phosphorus molybdenum blue and silicon molybdenum blue, respectively [23]. The nutrients were analyzed using a Skalar San++ continuous flow analyzer. The preparation of the working standards and the detection limits of nutrients were described in detail in our previous study [21]. 

### 2.4. Measurement of Carbon Fixation Rate (CFR)

The ^14^C tracer assay was used to determine the CFR using phytoplankton [24,25]. A culture solution of 0.01 μCi ^14^C mL^−1^ was produced by adding ^14^C-labeled NaHCO_3_ (1 μCi) to each sample. Duplicate samples were exposed to light and parallel cultivation was carried out under darkness. The phytoplankton were incubated for 4 to 6 h in an on-site incubator at a maintained temperature in flowing seawater at a depth of ~5 m. Micro-, nano-, and pico-phytoplankton were collected gently with a 10 μm polycarbonate filter, a 0.7 μm glass fiber filter, and a 0.2 μm polycarbonate filter in sequence [21]. Specific details such as the on-deck incubation and sample filtration procedures were described in our previous studies [21,26]. The filters containing ^14^C assimilated by phytoplankton were stored frozen until the analysis. The radioactivity of ^14^C was counted using a liquid scintillation counter (Tricarb 2900TR, Pekin Elmer, Turku, Finland). The CFR was calculated using the following equation [27]:

$$\mathrm{CFR}\;\left({\mathrm{mmol}\;C\;m}^{-3}d^{-1}\right)=\frac{\mathrm{DIC}\text{×}\left({\mathrm{dpm}}_{\mathrm{light}}-{\mathrm{dpm}}_{\mathrm{dark}}\right)\times 1.05}{{\mathrm{dpm}}_{\mathrm{total}}\times T}$$

where DIC is the content of dissolved inorganic carbon (mmol m^−3^); dpm_light_, dpm_dark_, and dpm_total_ are the disintegrations per minute of ^14^C under light culture, dark culture, and total addition conditions, respectively. The constant 1.05 is the discrimination factor between the incorporation of ^14^C and ^12^C [27], and T is the incubation time (d). 

### 2.5. Measurement of Iron Uptake Rate (FeUR)

Artificial Fe-free experiments were conducted to minimize any Fe contamination [21,26,28,29,30]. The filtered seawater was removed from the background Fe using a Chelex-100 column under a class-100 clean laminar flow hood. The collected algal cells were rinsed with EDTA–oxalate reagent to remove the extracellularly adsorbed Fe [30]. Algal cells with extracellular Fe removed were resuspended in Fe-free seawater in duplicate. The radiotracer ^55^Fe (Eckert & Ziegler, Wilmington, MA, USA, 104.3 mCi/mg Fe as FeCl_3_) was added and the FeUR rate was determined after incubation. After 24 h of on-deck culture, the phytoplankton samples were collected gently with a 10 μm polycarbonate filter, a 0.7 μm glass fiber filter, and a 0.2 μm polycarbonate filter in sequence to obtain micro-, nano-, and pico-phytoplankton samples, respectively [21]. The EDTA–oxalate regent was used to remove the extracellularly adsorbed Fe [30]. The filter containing intracellular Fe was stored frozen prior to the laboratory analysis. 

The radioactivity of the intracellular ^55^Fe was determined using a liquid scintillation counter (Tricarb 2900TR, Pekin Elmer) according to Wang et al.’s study [21]. The FeUR rate was calculated using the following equation:

$$\mathrm{FeUR}\;\left({\mathrm{pmol}\;\mathrm{Fe}\;L}^{-1}d^{-1}\right)=\frac{{\mathrm{dpm}}_{\mathrm{measured}}-{\mathrm{dpm}}_{\mathrm{background}}}{f\times V\times T}$$

where dpm_measured_ and dpm_backgound_ are the disintegrations per minute of ^55^Fe in the post-culture and initial samples, respectively. Here, *f* is the factor used to convert the radioactivity of ^55^Fe to the Fe content (pmol), which was 1472.8 in this study. V and T represent the incubation volume (0.5 L) and incubation time (1 d), respectively. 

The removal efficiency of extracellular Fe by the EDTA–oxalate reagent and the removal efficiency of DFe by the Chelex-100 resin was determined to ensure the Fe-free incubation experiments. Our preliminary results show that both Fe adsorbed in the extracellular fraction and DFe in seawater are effectively removed (>94%, Table S1). Therefore, our calculated FeUR accurately reflects the uptake rate of DFe by phytoplankton. Additionally, we determined the amount of ^55^Fe adsorbed on the filter and showed that only 2–4% of the added ^55^Fe was retained on the filter after the EDTA–oxalate reagent wash (Table S1). 

### 2.6. Determination of Mixed Layer Depth (MLD)

The water in the surface mixed layer has a nearly uniform temperature, density, and salinity. Changes in the MLD are important for phytoplankton growth [31,32,33,34]. The MLD was determined in this study using a combination of the following three methods: (1) the depth at which the potential temperature differs from the temperature at 10 m by 0.2 °C; (2) the depth at which the potential density is 0.03 kg m^−3^ higher than that at 10 m [35,36]; (3) the minimum depth for sudden changes in temperature and density profiles [33].

### 2.7. Quantification of Sea Ice Meltwater (SIM) and Meteoric Water (MW)

#### 2.7.1. Measurement of Stable Oxygen Isotopic Composition

The samples for the δ^18^O measurements were collected from the surface to the bottom according to the standard water strata (5, 25, 50, 75, 100, 200, 500, 1000, 2000, and 3000 m and near the bottom). The seawater was transferred to a 2 mL glass screw cap vial for the δ^18^O analysis. The measurement of δ^18^O in seawater was done using wavelength-scanning cavity ring-down spectroscopy in a laser spectroscopy analyzer (Picarro L2140-I, Santa Clara, CA, USA) as we previously described [21,37]. 

#### 2.7.2. Calculation of Freshwater Components (SIM and MW)

The SIM, MW, and CDW are considered the three components in the upper water of the AS. The fractions of these three components are estimated from the mass balance of δ^18^O and S with the following equations:

f_SIM_ + f_MW_ + f_CDW_ = 1

f_SIM_ × S_SIM_ + f_MW_ × S_MW_ + f_CDW_ × S_CDW_ = S

f_SIM_ × δ^18^O_SIM_ + f_MW_ × δ^18^O_MW_ + f_CDW_ × δ^18^O_CDW_ = δ^18^O

where f_SIM_, f_MW_, and f_CDW_ represent the fractions of SIM, MW, and CDW, respectively. S_SIM_, S_MW_, and S_CDW_ are the salinity levels of the three endmember waters, respectively. Here, δ^18^O_SIM_, δ^18^O_MW_, and δ^18^O_CDW_ are the δ^18^O values of the three endmember waters, respectively. S and δ^18^O represent the measured salinity and δ^18^O value of the water sample, respectively.

Table S2 lists the characteristic values of S and δ^18^O in the three endmember waters. The average salinity of the annual sea ice (7.0) was taken as the characteristic salinity of the SIM [38,39]. The δ^18^O value of the SIM (2.1‰) refers to the value in the freshwater study of the AS by Biddle et al. [40]. The salinity (0) and δ^18^O (−25.0‰) of the MW used in this study were cited from Randall-Goodwin et al. [41] and Biddle et al. [40]. The salinity and δ^18^O values of CDW were chosen to be 34.78 and 0.1‰, respectively, which were determined based on the measurements in our voyage and referring to Biddle et al. [40] and Randall-Goodwin et al. [41]. The scatter plot of δ^18^O versus S showed that most of the samples fell into the mixed region surrounded by the three endmembers, indicating that the values of δ^18^O and S for the three endmembers were appropriate (Figure S1). Some samples lay above or below the CDW-SIM line, showing the effects of the formation (F) or melting (M) of sea ice, respectively.

The uncertainty levels of f_SIM_ and f_MW_ calculated above are affected by the values of S and δ^18^O for the three endmembers, of which the δ^18^O of MW has the greatest influence. Meteoric water is a combination of glacial melt water and precipitation (including snow and rain), with different δ^18^O values. The reported δ^18^O values of the MW varied widely, from −40‰ [42] to −13‰ [43]. Here, we evaluate the uncertainty of our calculations by taking the lowest (−40‰) and highest (−13‰) values as the δ^18^O endmembers of the MW, respectively. When the δ^18^O values of the MW vary from −25‰ to −13‰, the relative deviation of the f_MW_ is between 1.2% and 2.8% (average 2.0%), and the relative deviation of the f_SIM_ is between −1.5% and −3.5% (average −2.5%). When the δ^18^O values of the MW vary from −40‰ to −25‰, the relative deviation falls between 0.8% and 1.5% (average 1.2%), and the relative deviation of the f_MW_ is between 1.0% and 1.9% (average 1.5%). Therefore, the uncertainty in our calculation of the f_MW_ and f_SIM_ was less than 3.5%. In addition, the errors caused by the uncertainty of the endmembers were systematic and had little effect on the relative fractions of the freshwater components.

### 2.8. Statistical Analyses

The results are presented as means ± the standard deviation (SD), and the figures were generated using OriginPro 8.5 and Ocean Data View 5.1.7 software. Differences between the two groups were compared using a one-way ANOVA (*p* = 0.05). Tukey’s post hoc test was used to test the hypothesized differences. The differences were qualified and then determined to be statistically significant (*p* ≤ 0.05). The relationship between the environmental factors and measured biological variables was tested using Spearman’s rank correlation coefficient with the corrplot R package (R version 3.6.0).

## 3. Results

### 3.1. Physical and Hydrochemical Properties

The physical and hydrochemical characteristics in the surface seawater at our sampling stations showed clear meridional differences, as shown in Figure S2. The surface water temperature (SST) values varied from −1.84 °C to 2.81 °C, with an average of −0.50 ± 1.85 °C. Spatially, the SST in the high-latitude stations were lower than −1 °C, with the lowest occurring at station A1-03 (−1.84 °C), while the SST in the northern stations (stations A2-15 and A2-17) was higher than 2 °C (Figure S2a). 

The surface water salinity (SSS) values ranged from 33.23 to 34.04, with an average of 33.54 ± 0.27, and the lowest appeared at station A1-04. The SSS at the northernmost stations (stations A2-15 and A2-17) was greater than 33.90, which was higher than that of the other stations (Figure S2b). 

The σ_0_ values in our study sites varied from 26.75 to 27.19 kg m^−3^, with an average of 26.93 ± 0.14 kg m^−3^. The highest potential density appeared at station A2-15, the lowest appeared at station A1-04, and the value was also lower at station A1-03 (26.77 kg m^−3^) (Figure S2c). The temperature, salinity, and density of the surface water at stations A1-03 and A1-04 were lower, indicating that they were more affected by glacial meltwater or sea ice meltwater than other stations. 

The dissolved oxygen (DO) concentrations in the surface water ranged from 307.52 to 356.59 μmol L^−1^, with an average of 340.90 ± 14.72 μmol L^−1^. Spatially, the DO of the surface water in the 68° S to 73.2° S region (>343.65 μmol L^−1^) was more oxygen-rich than that north of 68° S, reflecting the increased solubility caused by the lower SST (Figure S2d). 

The mixed layer depth (MLD) values at our study stations varied from 26 m to 137 m, with an average of 54 ± 32 m, with the stations A2-17 being the deepest and A2-02 the shallowest. Overall, the MLD values gradually became shallower with increasing latitude, with the MLD in the high latitudes from 68° S to 73.2° S being less than 44 m and greater than 51 m in the northern regions (Figure S2e). 

The depths of the maximum chlorophyll layer (CMD) at our study stations ranged from 1 m to 79 m, with an average of 27 ± 23 m. The deepest CMD occurred at station A2-15, while the CMD values at stations A1-02 and A2-04 were 1 m and 3 m, respectively. Stations A2-02, A2-11, and A2-09 also had shallower CMDs (13–17 m) (Figure S2f). 

The dissolved inorganic nitrogen concentration (DIN) values in the surface seawater changed from 17.94 to 26.90 μmol L^−1^, with an average of 20.91 ± 0.86 μmol L^−1^, showing an increase to the north (Figure S2g). The lower DIN in the southern high latitudes reflected the utilization of nutrients by phytoplankton in the late algal bloom. Contrary to the overall low DIN at high latitudes, station AD-02 exhibited the highest DIN, corresponding to a low phytoplankton biomass (fluorescence intensity <0.26 μg L^−1^). In addition, the higher DIN at stations A2-15 (23.66 μmol L^−1^) and A2-17 (23.31 μmol L^−1^) may be related to less nutrient depletion due to low biomass or nutrient replenishment by CDW upwelling (Figure S2g).

The fluorescence intensity values in the surface seawater in the AS ranged from 0.14 μg L^−1^ (station A2-08) to 7.80 μg L^−1^ (station A1-02). Notably, the fluorescence intensity values in the surface seawater at the southernmost (AD-02: 0.26 μg L^−1^) and northernmost stations (A2-17: 0.40 μg L^−1^) were lower, while the fluorescence intensity at station A2-11 was higher (7.40 μg L^−1^) (Figure S2h). As shown by the distribution of Chl *α* in the surface water in Figure S2i, the Chl *α* was generally higher in the surface seawater in high-latitude regions (>65° S) than in northern regions. In fact, the fluorescence intensity showed a significant positive correlation with Chl *α* in the surface seawater (r = 0.93, *p* < 0.01, Figure S3), indicating that the fluorescence intensity well reflected the changes in phytoplankton biomass in our study area.

### 3.2. δ^18^O in Seawater and Fraction of Freshwater Components

**Figure 2:**
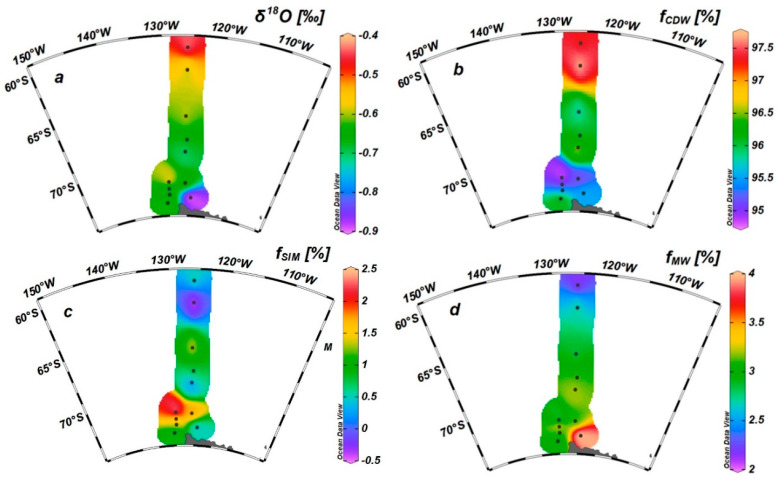
Spatial distribution of δ18O (‰) (**a**) and fractions of circumpolar deep water (CDW) (%) (**b**), sea ice meltwater (%) (**c**), and meteoric water (%) (**d**) in surface water.

The δ^18^O values in the surface seawater ranged from −0.89‰ to −0.43‰, with an average of −0.64‰ ± 0.11‰. The δ^18^O in the surface water at station A2-17 was the highest, while station A2-02 was the lowest. In general, the δ^18^O in the northern region was higher than that in the coastal region of the Antarctic continent (Figure 2a). 

The fractions of sea ice meltwater (f_SIM_) ranged from −0.42% (station A1-04) to 2.34% (station A2-02) with an average of 0.99% ± 0.78%. Note that the f_SIM_ values in the surface and subsurface water samples of some stations were negative, such as at 50 m at station A2-02, 40 m at station A2-08, and 0 and 25 m at station A2-15, showing the effect of the brine released by sea ice formation (Figure 2c). This was consistent with the formation of lotus leaf ice observed during the field survey. The fractions of MW ranged from 2.14% (station A2-17) to 3.99% (station A2-02), with an average of 3.03% ± 0.45%, showing an overall decrease from nearshore to offshore (Figure 2d). 

The changes in the fraction of total freshwater (f_SIM_ + f_MW_) in surface seawater were closely related to changes in salinity and potential density. With the increasing freshwater fractions, the salinity and potential density decreased (*p* < 0.001, Figure S4a). Moreover, the MLD and CMD became shallower with the increasing freshwater fractions (*p* < 0.05, Figure S4b). Due to the mechanism by which sea ice and glaciers are formed, ice meltwater is less saline and less dense than circumpolar deep water. The input of fresh water will reduce the salinity and density of the surface seawater, leading to a shallower mixed layer and increased water stability, thereby promoting phytoplankton growth and the formation of a maximum chlorophyll layer. 

### 3.3. CFR and FeUR

**Figure 3:**
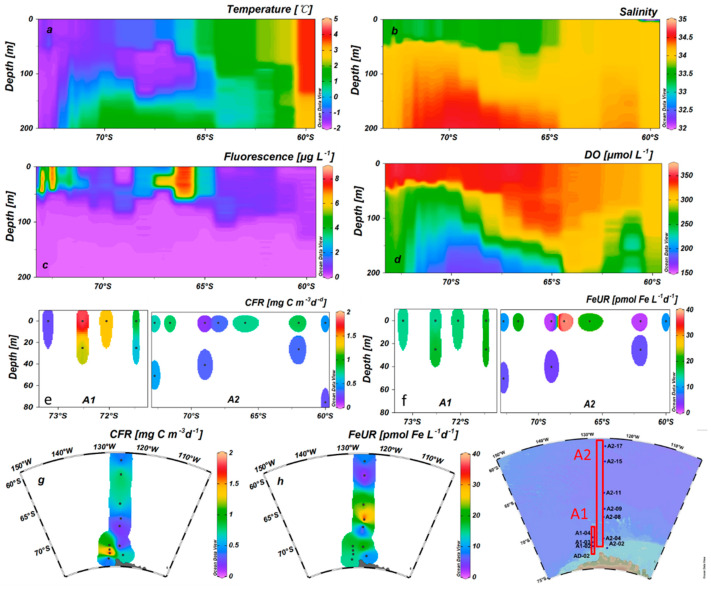
The distribution of the temperature (°C) (**a**), salinity (psu) (**b**), fluorescence (μg L^−1^) (**c**), DO (μmol L^−1^) (**d**), CFR (mgC m^−3^ d^−1^) (**e**), and FeUR (pmolFe L^−1^ d^−1^) (**f**) values in sections A1 and A2 and the CFR (mgC m^−3^ d^−1^) (**g**) and FeUR (pmolFe L^−1^ d^−1^) (**h**) distributions in the surface water. Note that only CFR and FeUR in the surface and CMD layers were determined.

The CFR values in the surface seawater varied from 0.16 to 1.83 mgC m^−3^ d^−1^ (mean 0.70 ± 0.53 mgC m^−3^ d^−1^), with the highest occurring nearshore (station A1-02) and the lowest occurring at station A2-08 (Figure 3e,g, Table 1). The highest value of CFR in the surface water at section A2 appeared at station A2-11, corresponding to a high phytoplankton biomass (Figure S2h,i). The CFR values in the subsurface layer ranged from 0.29 to 1.34 mgC m^−3^ d^−1^, with an average of 0.55 ± 0.41 mgC m^−3^ d^−1^. The highest CFR value in the subsurface occurred at station A1-02, the same as the station with the highest surface value, while the lowest appeared at station A2-15 (Figure 3e, Table 1). Overall, the CFRs in the subsurface layer were lower than those on the surface, except for station A2-08. The low CFR in the surface water of station A2-08 was related to the extremely low biomass. In the spatial comparison, the CFR in the AS was an order of magnitude lower than that of the eastern Antarctic Peninsula (1.8–21.48 mgC m^−3^ d^−1^, [21]). 

The FeUR rates by the phytoplankton in the surface seawater ranged from 1.66 to 38.19 pmolFe L^−1^ d^−1^, with an average of 14.96 ± 10.18 pmolFe L^−1^ d^−1^. The FeUR was generally higher for the shelf stations close to the Antarctic continent, although the highest value occurred at station A2-09. The lowest FeUR occurred at station A2-15, while its adjacent station A2-11 was higher (21.88 pmolFe L^−1^ d^−1^) (Figure 3f, Table 1). The FeUR in the subsurface values ranged from 4.58 to 20.29 pmolFe L^−1^ d^−1^, with a mean of 10.73 ± 7.57 pmolFe L^−1^ d^−1^. The highest value appeared at station A1-04 and the lowest appeared at station A2-08. Similar to the spatial variation in the surface layer, the FeUR values in the subsurface layer were generally higher at the shelf stations, possibly related to the CDW upwelling providing more DFe. The sectional distributions of temperature, salinity, and DO indicated that the CDW with high salinity and low DO affected the shelf area close to the Antarctic continent (Figure S2a,b,d and Figure 3a,b,d). Unlike most shelf stations, the FeUR at station A2-02 was low (0 m: 9.53 pmolFe L^−1^ d^−1^; subsurface: 5.55 pmolFe L^−1^ d^−1^), corresponding to a low fluorescence intensity (phytoplankton biomass). Except for station A2-02, the FeURs in the subsurface layer were generally higher than those in the surface layer. The FeUR in the AS was close to that of the surface waters in the eastern Antarctic Peninsula (5.16–16.31 pmolFe L^−1^ d^−1^, average: 10.81 ± 4.26 pmolFe L^−1^ d^−1^, [21]) and the Antarctic polar front (1.20–14.40 pmolFe L^−1^ d^−1^, [44]), but lower than that of the sub-Antarctic waters (540 pmolFe L^−1^ d^−1^, [44]; 85.68 pmolFe L^−1^d^−1^, [45]). 

**Figure 4:**
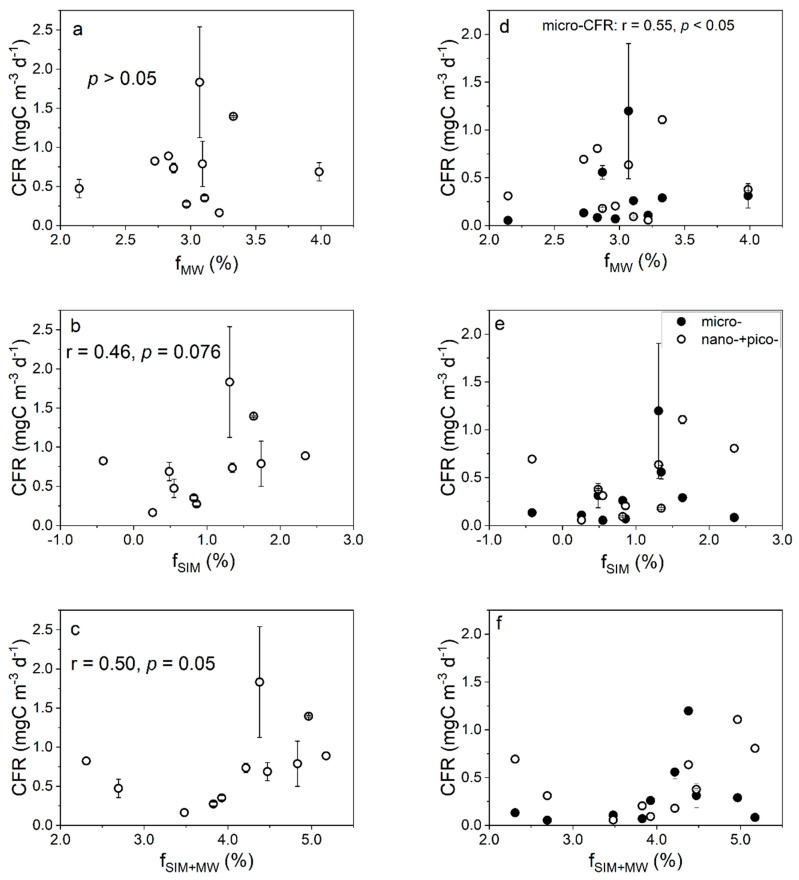
The relationship between the CFR (left panel) and size-fractionated CFR (right panel) values and the f_MW_ (**a**,**d**), f_SIM_ (**b**,**e**), and f_SIM+MW_ (**c**,**f**) values in the surface water. The solid and open circles represent the micro-CFR values and the sum of nano-CFR and pico-CFR values, respectively.

**Figure 5:**
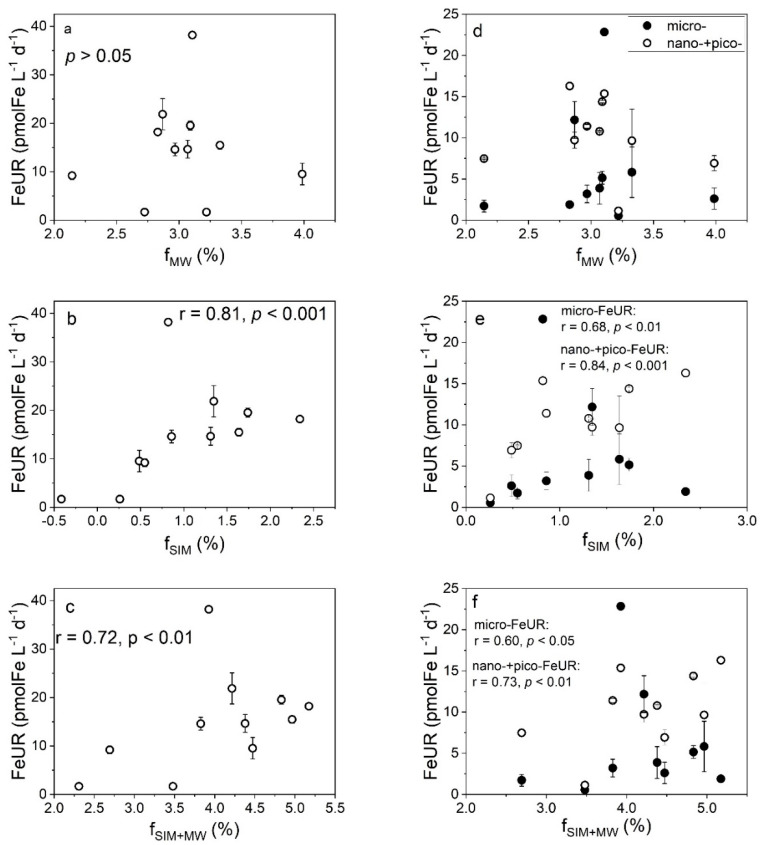
The relationship between the FeUR (left panel) and size-fractionated FeUR (right panel) values and f_MW_ (**a**,**d**), f_SIM_ (**b**,**e**), and f_SIM+MW_ (**c**,**f**) values in the surface water. The solid and open circles represent the micro-FeUR values and the sum of nano-FeUR and pico-FeUR values, respectively.

As shown in Figure 4a–c, a significant positive correlation (*p* = 0.05) was observed between the CFR and total freshwater fraction (f_MW_ + f_SIM_), but not between the CFR and f_SIM_ or f_MW_. The FeUR was not significantly correlated with the f_MW_ (*p* > 0.05, Figure 5a) but was positively correlated with the f_SIM_ (r = 0.81, *p* < 0.001, Figure 5b), indicating that the FeUR in the Amundsen Sea is more affected by sea ice meltwater. Despite the differences in the effects of meteoric water and sea ice meltwater on the FeUR, the total freshwater input promoted the FeUR in the Amundsen Sea, as evidenced by their significant positive correlation (*r* = 0.71, *p* < 0.01, Figure 5c).

### 3.4. Size-Fractionated CFR and FeUR

**Figure 6:**
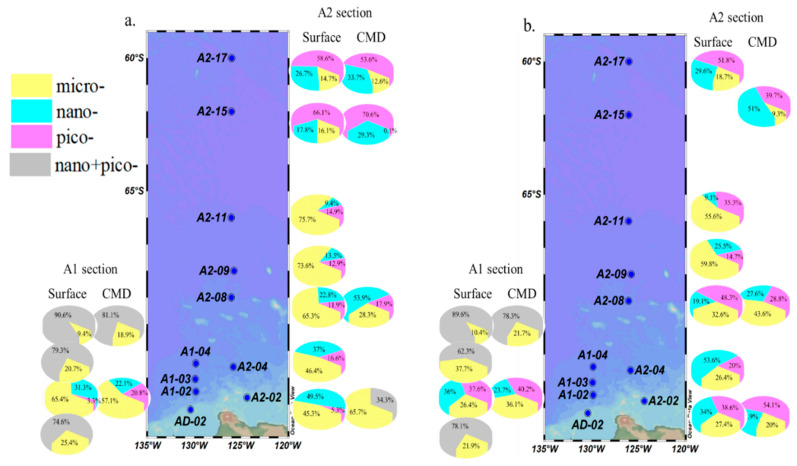
The contributions of the size-fractionated CFR (**a**) and FeUR (**b**) to the total CFR and FeUR. The left and right sides outside the sampling map represent sections A1 and A2, respectively. Different colors represent the contributions of phytoplankton of different sizes, where yellow represents micro-plankton, light blue represents nano-plankton, pink represents pico-plankton, and grey represents nano- and pico-plankton, respectively.

The contributions of the micro-phytoplankton (micro-CFR), nano-phytoplankton (nano-CFR), and pico-phytoplankton (pico-CFR) to the total CFR exhibited significant spatial variation (Figure 6a). The contribution of the micro-CFR to the total CFR varied from 0.1% to 75.7%, with <17% being found in the open ocean and >50% in other sites. The lowest contribution of the micro-CFR occurred at 25 m at station A2-15 in the open sea (0.1%), while high contributions appeared at stations A2-11 (0 m: 75.7%), A2-09 (0 m: 73.6%), A2-08 (0 m: 65.3%), and A1-02 (0 m: 65.4%, 50 m: 57.1%). The contribution of the nano-CFR to the total CFR ranged from 9.4% to 53.9%, with the highest occurring at 40 m at station A2-08 and the lowest at 0 m at station A2-11. The spatial variation of the nano-CFR contributions was similar to that of the micro-CFR. The contributions of the pico-CFR to total CFR ranged from 3.3% to 70.6%, with higher values being found in the open ocean. The pico-CFR contribution was highest at station A2-15 (0 m: 66.1%, 25 m: 70.6%), followed by station A2-17 (0 m: 58.6%, 75 m: 53.6%), while the other stations were <25%, especially stations A1-02 (3.3%) and A2-02 (5.3%). In terms of the contributions of the three sizes of phytoplankton to the CFR, the micro-phytoplankton contributed the most, while the nano-phytoplankton and pico-phytoplankton contributed similarly. 

The contribution of the fractionated phytoplankton to the total FeUR is shown in Figure 6b. The contributions of the micro-FeUR to the total FeUR ranged from 9.3% to 59.8%, with >50% being found at stations A2-09 and A2-11, corresponding to a high proportion of micro-phytoplankton biomass. The contributions of the nano-FeUR to the total FeUR ranged from 9.1% to 53.6%, with the highest being found at 0 m at station A2-04 (53.6%), followed by 25 m at station A2-15 (51%), and the lowest being found at 0 m at station A2-11 (9.1%). The contributions of the pico-FeUR to the total FeUR ranged from 14.7% to 54.1%, with the highest being found at the subsurface at station A2-02 and the lowest at the surface at station A2-09. On average, the contributions of the three sizes of phytoplankton to the FeUR were not obviously different, with the pico-FeUR contribution being slightly higher than the other two larger phytoplankton sizes. 

As shown in Figure 4d–f, the relationship between the size-fractionated CFR and freshwater components showed that the micro-CFR increased with the increasing f_MW_ values (r = 0.55, *p* < 0.05, Figure 4d), but the nano- + pico-CFR was not affected by the f_MW_ and there was no significant correlation between the size-fractionated CFR and the f_SIM_ or f_MW + SIM_ (Figure 4e,f). Similarly, the contribution of the micro-CFR to the CFR increased significantly with the increasing f_MW_ values (*p* < 0.05, Figure S5a), while the contribution of the nano- + pico-CFR decreased (*p* < 0.05, Figure S5a) and the contribution of the size-fractionated CFR was not affected by the f_SIM_ and f_MW+SIM_ (*p* > 0.05, Figure S5b,c).

As shown in Figure 5d–f, the relationship between the size-fractionated FeUR and freshwater components was similar to that between the total FeUR and freshwater components. The size-fractionated FeUR was not significantly correlated with the f_MW_ (Figure 5d) but was significantly positively correlated with the f_SIM_ and f_SIM+MW_. In particular, both the micro-FeUR and nano- + pico-FeUR increased with the increasing f_SIM_ and f_SIM+MW_ values (Figure 5e,f). The contributions of the size-fractionated FeUR to total the FeUR varied by cell size. With the f_MW_ values increasing, the contribution of the micro-FeUR increased slightly (r = 0.39, *p* = 0.08), while that of the nano- + pico-FeUR decreased (Figure S5d). Unlike the effect of the meteoric water, changes in the f_SIM_ and f_SIM+MW_ values did not affect the contribution of the size-fractionated FeUR to the total FeUR (Figure S5e,f).

### 3.5. Effects of Fe Enrichment on CFR and FeUR

**Figure 7:**
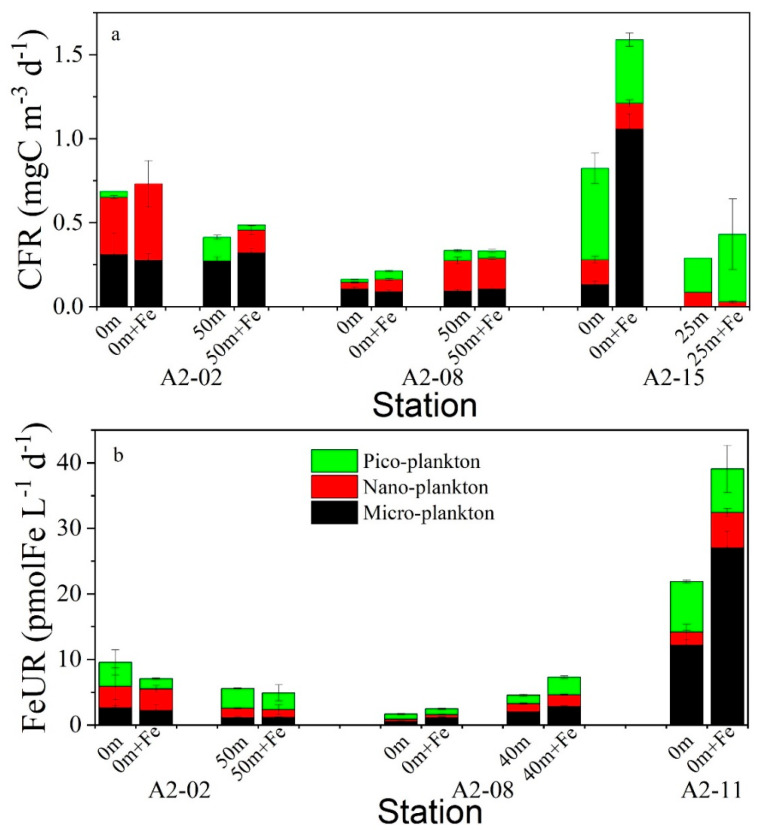
Effects of the DFe addition on the size-fractionated CFR (**a**) and FeUR (**b**). The black, yellow, and green bars represent micro-plankton, nano-plankton, and pico-plankton, respectively.

The CFR responses of the size-fractionated phytoplankton with and without Fe addition are shown in Figure 7a. The CFRs in the surface and CMD layers at the coastal station A2-02 did not respond to the Fe addition, while the contributions of the nano-CFR increased after the DFe addition. The DFe addition had different effects on the CFR in the surface and CMD layers at station A2-08. The DFe addition in the surface water slightly increased the CFR values from 0.16 mg C m^−3^ d^−1^ to 0.21 mg C m^−3^ d^−1^, mainly contributed by the nano- and pico-phytoplankton, while the DFe addition had no obvious effect on the CFR in the CMD water. The situation at station A2-15 was somewhat similar to that at station A2-08. The CFR in the surface water was distinctly increased after the DFe addition (from 0.82 mg C m^−3^ d^−1^ to 1.59 mg C m^−3^ d^−1^), with the micro-phytoplankton being the most obvious, while the CFR in the CMD layer (25 m) had no obvious response to the DFe addition. The differences in the responses of the CFRs to the DFe addition showed the effects of the Fe on the phytoplankton samples of different sizes in the different sites. The addition of the DFe had no significant effect on the CFRs at coastal station A2-02, indicating that the growth of phytoplankton may not be limited by Fe, but may be more affected by light intensity. Unlike the Antarctic coastal ocean, the growth of the phytoplankton at station A2-15 in the open ocean affected by the Antarctic Circumpolar Current may be limited by Fe availability, resulting in a significant increase in the micro-CFR and its contribution after the addition of DFe. Compared with micro-phytoplankton, nano- and pico-phytoplankton are less affected by Fe limitations because smaller cells have a competitive advantage in Fe uptake due to their larger specific surface area. The response of the phytoplankton to the Fe addition at station A2-08 was intermediate between stations A2-02 and A2-15. Note that the CFR in the CMD layer at station A2-08 was higher than that in the surface layer, regardless of the DFe addition, possibly related to changes in the phytoplankton biomass or community structure. 

The FeUR responses of size-fractionated phytoplankton with and without Fe addition are shown in Figure 7b. Similar to the response of the CFR, the addition of DFe had no significant effect on the FeUR of phytoplankton of different sizes in the surface and CMD layers at station A2-02. Unlike station A2-02, the DFe addition distinctly increased the FeUR in the surface and CMD layers at station A2-08, where the FeUR increased from 1.68 pmolFe L^−1^ d^−1^ to 2.47 pmolFe L^−1^ d^−1^ at 0 m and from 4.58 pmolFe L^−1^ d^−1^ to 7.27 pmolFe L^−1^ d^−1^ at 40 m. The phytoplankton at station A2-11 also showed a positive response to the Fe addition, which was mainly contributed by micro-phytoplankton. The micro-FeUR increased from 12.17 ± 2.25 pmolFe L^−1^ d^−1^ to 27.01 ± 2.52 pmolFe L^−1^ d^−1^ after the DFe addition, and its contribution to the FeUR increased from 55.6% to 69.1%. The responses of the FeUR to the DFe addition at the three stations showed that the FeUR at the coastal station A2-02 was not affected by Fe addition, while the FeUR at the offshore stations A2-08 and A2-11 increased after the addition of DFe. These different responses may have been related to the initial DFe content in the seawater. It is worth noting that at station A2-08, the Fe addition did not affect the CFR but the FeUR was elevated, possibly implying excess Fe uptake by the phytoplankton. At station A2-11, the increase in FeUR after the Fe addition was mainly caused by the micro-phytoplankton, with little change in the nano-FeUR and pico-FeUR. This indicates that phytoplankton with larger cell size are more severely limited by DFe, and nano- and pico-phytoplankton with smaller cell sizes have the advantage of Fe absorption due to their larger specific surface area, meaning they are less affected by Fe deficiencies. 

## 4. Discussion

The stability and DFe concentration in seawater are considered to be two important factors affecting the primary productivity in the SO and other HNLC waters [10,14,46,47,48,49]. In the SO, the sea ice meltwater and meteoric water not only enhance the stability of the seawater [1], but also provide sources of iron for phytoplankton growth [13,50]. Previous studies on the Antarctic Peninsula have shown that the enhanced water stability caused by the input of meteoric water and sea ice meltwater increases the CFR and FeUR in the summer [21]. How the situation in the Amundsen Sea is affected will require further studies.

### 4.1. Effects of Freshwater Components on the CFR

**Figure 8:**
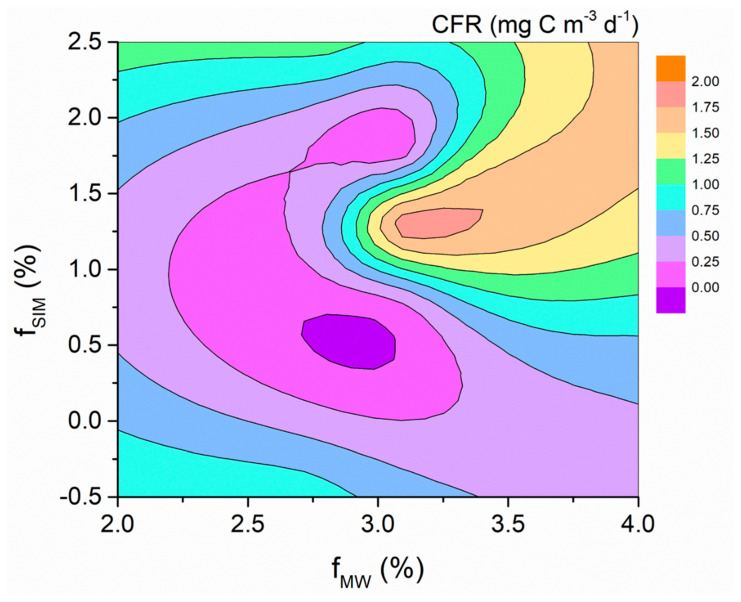
The CFR variations with the fractions of MW (f_MW_) and SIM (f_SIM_).

**Figure 9:**
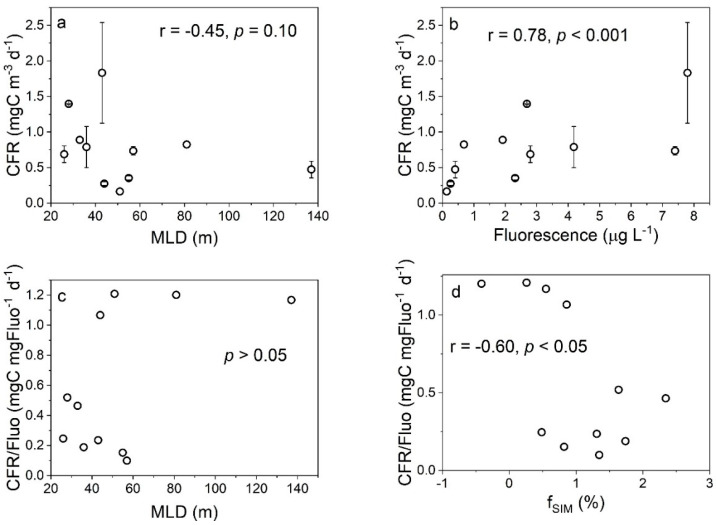
The relationships between the CFR and the MLD (**a**) and phytoplankton biomass (expressed as fluorescence) (**b**) and between the biomass-normalized CFR and the MLD (**c**) and f_SIM_ (**d**) in surface water.

The relationships between the CFR and the f_MW_ and f_SIM_ indicated that the higher CFRs in the AS appeared in the regions where both meteoric water and sea ice meltwater were abundant, indicating that the input of meteoric water and sea ice meltwater was beneficial to the photosynthesis of the phytoplankton (Figure 8). The total freshwater content has an effect on the carbon fixation in the AS, and the mechanisms for the effects of meteoric water and sea ice meltwater may be different (Figure 4a–c). The effect of the total freshwater on the CFR may be attributed to two reasons. First, the freshwater input enhances the water stability, which alleviates the light limitation and promotes carbon fixation [48,49]. Second, the freshwater input provides DFe, which relieves the Fe limitation and promotes carbon fixation [51,52]. Note that the spatial variation of the meteoric water during our sampling period was small, while that of the sea ice meltwater was large, and the lotus leaf ice formed south of 70° S (Figure 2). Therefore, the role of sea ice meltwater in regulating the CFR in the AS may be more important than that of meteoric water. The formation and melting of sea ice affects the CFR through two pathways that alter the DFe content and the distribution of phytoplankton in seawater. During sea ice formation, DFe is adsorbed onto ice crystals, resulting in a decrease in DFe. The adsorbed Fe is released back into the seawater when the sea ice melts, thereby increasing the CFR [53,54]. The formation and melting of sea ice also cause changes in the phytoplankton biomass. When sea ice is formed, phytoplankton are actively or passively incorporated into the ice core and are gradually released during the melting process of the sea ice, thereby “seeding” the blooms in the marginal ice zone [55,56]. Our results show that there is no significant correlation between the CFR, CFR/fluoresence ratio, and MLD, but there are significant correlations between the CFR and fluorescence and between the CFR/fluoresence ratio and f_SIM_ (Figure 9). This shows that in terms of the impact of the sea ice meltwater, changes in water stability are not the main factor affecting the CFR, but rather changes in phytoplankton biomass area. The spatial variation of the CFRs in the AS showed that higher CFRs appeared at stations with MLD values < 50 m and fluorescence intensity values >7 μg L^−1^ (Figure S6), also illustrating that the CFR was mainly affected by the phytoplankton biomass. However, the negative correlation between the CFR normalized to the phytoplankton biomass and the f_SIM_ suggests that sea ice melt leads to a decrease in CFR per unit biomass (*r* = −0.60, *p* < 0.05, Figure 9d), implying that the “seeding” effect of the sea ice melt may be more important than the stimulus effect. The weak correlation between the phytoplankton biomass and sea ice meltwater (*p* = 0.06, Figure S7b) but not meteoric water (*p* = 0.23, Figure S7a) may be because ice algae released from sea ice melt increase the phytoplankton biomass in the ice marginal zones. In addition, the more significant correlation between the phytoplankton biomass and freshwater input (f_MW_ + f_SIM_) (r = 0.59, *p* < 0.05, Figure S7c) suggests that sea ice melting may affect phytoplankton communities through several other pathways, such as DFe supply and light alleviation. 

These relationships between the size-fractionated CFR and freshwater components (Figure 4d–f and Figure S5a–c) suggest that the input of meteoric water is beneficial to improve the photosynthesis of micro-phytoplankton, affecting the community structure of the phytoplankton, while the sea ice meltwater promotes the CFR but has no significant impact on the phytoplankton’s community structure. The micro-phytoplankton are susceptible to Fe deficiency due to their low specific surface area. The freshwater inflow, especially the meteoric water input, is beneficial for enhancing the photosynthesis of micro-phytoplankton, thereby increasing the contribution of the micro-CFR. The relationship between the CFR and MLD suggests that the micro-CFR decreases with the increase in MLD (*r* = −0.53, *p* < 0.05), while the nano-+pico-CFR is not affected by changes in MLD (Figure S8a). The contribution of the size-fractionated CFR was not significantly correlated with the MLD (Figure S8b). For stations with MLD values < 75 m, the contribution of the micro-CFR increased with increasing MLD and the contribution of nano-+pico-CFR decreased. This was because light may have played a more important role. As the MLD deepened, the algal cells of the nano-+pico-phytoplankton experienced a greater “shading effect” than the micro-phytoplankton. For stations with MLD values > 75 m, the contribution of the nano-+pico-CFR was much greater than that of the micro-CFR. The above relationships suggest that the increased MLD may favor the photosynthesis of smaller phytoplankton, as nano- and pico-phytoplankton adapt to Fe-limited environments. In addition, the relatively deeper MLD values compared to spring and summer [19] also indicated seasonal variation in the phytoplankton production in the AS.

### 4.2. Effects of Freshwater Components on the FeUR

**Figure 10:**
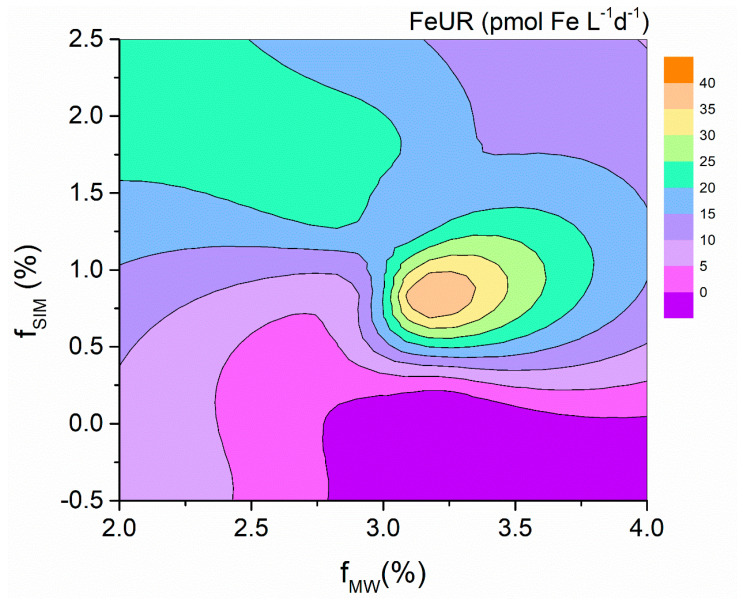
The FeUR variations with the f_MW_ and f_SIM_.

The high FeURs occurred at stations with f_SIM_ values > 0.75% and f_MW_ values of around 3.2% (Figure 10), which was roughly the same area as for the high CFRs. Similar to the CFR, the FeUR in the Amundsen Sea is more strongly influenced by sea ice meltwater (Figure 5a–c). This is different from the case where the FeUR is mainly affected by the meteoric water in the eastern Antarctic Peninsula [21] and some other SO waters [13,38,57,58]. The reason for the difference may be related to the different sampling locations and dates. The sampling period of this study was in the late summer and early autumn, when the spatial variation of the f_MW_ was small, while the f_SIM_ was large, which led to a greater impact of the sea ice meltwater on the FeUR. In general, the melting of sea ice and glaciers in late spring and early summer replenishes the DFe in seawater and promotes algal blooms in coastal waters. However, in autumn, similar to this study, the DFe is depleted during the early stages of phytoplankton growth, and the light limitation due to sea ice formation suppresses the phytoplankton growth, resulting in a low autumn CFR. The difference in DIN utilization rates by phytoplankton, such as the lower DIN concentrations in the AS than in the eastern Antarctic Peninsula (20.42–37.25 μmol L^−1^, [21]), also suggests seasonal variations in phytoplankton growth in the SO.

**Figure 11:**
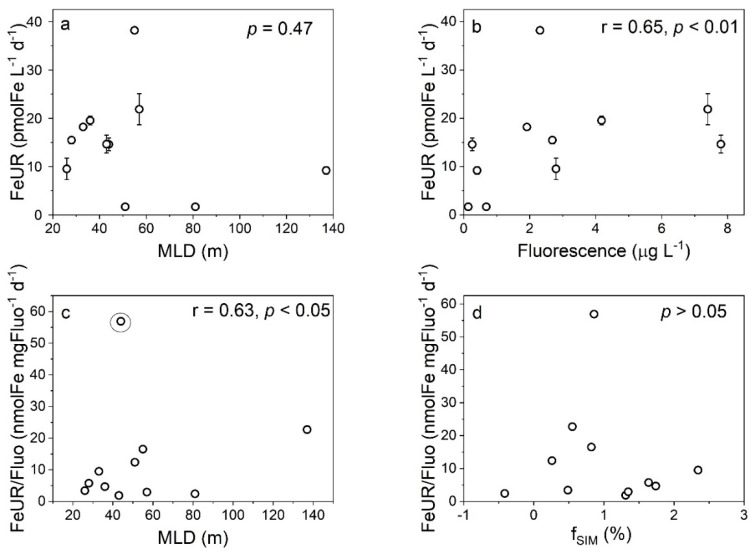
The relationships between the FeUR and the MLD (**a**) and phytoplankton biomass (expressed as fluorescence (**b**) and between the biomass-normalized FeUR and the MLD (**c**) and fSIM (**d**).

We did not observe a significant correlation between the FeUR and MLD in this study (Figure 11a), which may be related to the fact that the phytoplankton uptake of DFe occurs in weak light or darkness [21,59]. Considering that the light intensity has a limited effect on the FeUR, the weakening of the incident light intensity caused by the sea ice formation may have little effect on the FeUR, but the melting of sea ice leads to the stimulation of the FeUR due to the release of DFe or phytoplankton (the “seeding effect”). In fact, the FeUR is significantly positively correlated with the fluorescence intensity (*r* = 0.65, *p* < 0.01, Figure 11b), indicating that the phytoplankton biomass affects the FeUR in the AS. Noting that the phytoplankton biomass in the AS is affected by the sea ice meltwater (Figure S7b), the effects of the sea ice meltwater on the FeUR may be related to changes in phytoplankton biomass. Our results show that the FeUR normalized to the biomass is clearly positively correlated with the MLD (except station AD-02), indicating that an increase in MLD helps to increase the FeUR per unit of biomass in the late summer and early fall (Figure 11c). Unlike the MLD, the sea ice meltwater has no significant effect on the FeUR per unit of biomass (Figure 11d), possibly reflecting the “seeding effect”, i.e., the dilution of cell division to the normalized FeUR weakens the effect of the DFe released by sea ice melting. 

The relationships between the size-fractionated FeUR and the freshwater components (Figure 5d–f and Figure S5d–f) suggest that the input of meteorite water may alter the size structure of phytoplankton communities, but sea ice meltwater does not. The response of the size-fractionated FeUR to meteoric water and sea ice meltwater is similar to that of the CFR. The increase in meteoric water leads to an increase in the contribution of the micro-CFR, which correspondingly increases the demand for Fe by the micro-phytoplankton, thereby increasing the contribution of the micro-FeUR. The different effects of meteoric water and sea ice meltwater on the contribution of the size-fractionated FeUR may be related to the different influence mechanisms between these two freshwater components, for reasons that need to be further studied.

The change in MLD showed no significant effect on the size-fractionated FeUR (Figure S8c), further confirming that the stratification had little effect on the biological uptake of Fe in the AS. The contribution of the size-fractionated FeUR to the total FeUR had no significant relationship with the MLD, except for stations with MLDs < 75 m. In relatively stabilized waters (MLDs < 75 m), the contribution of the micro-FeUR increased with the increase in MLD, while the contribution of the nano- + pico-FeUR decreased accordingly (Figure S8d), which was similar to the changes in the size-fractionated CFR contribution. The reason may be that small-sized phytoplankton are less susceptible to Fe deficiencies, while MLD shows a more obvious stimulating effect on the uptake of Fe by large-sized phytoplankton.

### 4.3. Phytoplankton Demands for Fe and C

Table 2 lists the Fe/C ratios of phytoplankton assimilation in the surface and CMD layers of the AS, which range from 24 to 1301 μmol/mol, with an average of 329 ± 358 μmol/mol, well above the reported values in the eastern Antarctic Peninsula (2–34 μmol/mol, [21]), the sub-Antarctic waters (1.6 μmol/mol, [45]), the Bransfield Strait (17–26 μmol/mol, [60]), and the coastal western Antarctic Peninsula (32–53 μmol/mol, [61]). The higher Fe/C ratios in the AS may be related to the sampling time in the austral late summer and early autumn. Sea ice had begun to form in the sampling area at the time of sampling, when the carbon fixation was limited by light, while the absorption of Fe was not [62,63]. In low-light or even lightless conditions, the phytoplankton could absorb DFe from the ambient seawater for cellular growth and metabolism and could participate in a series of physiological cellular processes such as cellular respiratory electron chains, nitrate/nitrite reduction, the detoxification of reactive oxygen species, or storage in cells for photosynthesis to address iron deficiencies [62,64,65,66]. The different effects of light on carbon fixation and Fe uptake led to an increase in the Fe/C ratio in the AS.

The Fe/C ratios of phytoplankton at different sites responded differently to Fe enrichment (Table 2). In most stations, such as AD-02, A1-02, A1-03, A2-08, A2-09, and A2-11, the Fe addition led to an increase in the Fe/C ratio, with the coastal stations AD-02 (0 m) and A1-02 (0 m and CMD) being particularly significant. The Fe/C ratio of the phytoplankton in the surface water at station AD-02 increased by a factor of 2.8 from the initial 640 μmol/mol to 1783 μmol/mol after the addition of DFe. At station A1-02, after adding DFe, the Fe/C ratio in the surface layer increased from 96 μmol/mol to 1972 μmol/mol and that in the CMD layer increased from 171 μmol/mol to 972 μmol/mol, respectively, which were increases of 20.5 times and 5.7 times, respectively. Unlike the stations above, the Fe/C ratios at stations A1-04, A2-02, and A2-15 decreased by 10% to 71% after the addition of DFe. The causes of the different responses may have been due to differences in DFe concentrations or phytoplankton community structures in seawater. The relatively high concentration of DFe in the ambient environment may have been one of the reasons why the stimulating effect of the Fe enrichment was not obvious. In addition, phytoplankton communities dominated by large algal cells are more sensitive to Fe addition than those dominated by small algal cells.

The Fe enrichment has different effects on the Fe/C ratio in the phytoplankton of different sizes. The addition of DFe increased the Fe/C ratios of the micro-phytoplankton at most stations (e.g., AD-02, A1-02, A1-03, A2-08, A2-09, and A2-11), while the nano-+pico-phytoplankton were stimulated at only three stations (AD-02, A1-02, A1-03) (Table 2), indicating a more pronounced response of the micro-phytoplankton to the DFe addition. In addition, the initial Fe/C ratio of the micro-phytoplankton at stations A1-02, A2-08 (0 m), A2-09, and A2-11 was lower than that of the nano+ pico-phytoplankton, but the Fe/C ratio of the micro-phytoplankton after the Fe enrichment was higher than that of the nano-+pico-phytoplankton (Table 2), which also proved that the response of the micro-phytoplankton to the Fe enrichment was more significant. Two factors may have been responsible for the different responses of the size-fractionated phytoplankton. First, micro-phytoplankton are more susceptible to Fe limitations than nano- and pico-phytoplankton because of their larger cell size and smaller specific surface area. Secondly, micro-phytoplankton such as most diatoms usually absorb more Fe than their cellular metabolism needs when DFe is abundant, resulting in an increase in the cellular Fe/C ratio. Iron fertilization experiments in HNLC regions such as the Southern Ocean and the equatorial Pacific Ocean have found that diatoms with larger cells proliferate preferentially after Fe fertilization, while the biomass of smaller phytoplankton does not change significantly, indicating that the larger phytoplankton are more susceptible to Fe limitations [5,67]. In addition, laboratory culture experiments by Sunda and Huntsman [3,64] showed that *Thalassiosira weissflogii* and *Thalassiosira pseudonana* took up excess Fe in DFe-rich environments, with Fe/C ratios of 20–30 times that required for maximum cellular growth.

## 5. Conclusions

Global warming has accelerated the retreat of glaciers and sea ice in parts of Antarctica, leading to increased freshwater inputs into coastal areas of the SO. Our study shows that sea ice meltwater has a more pronounced effect on the CFR and FeUR in the Amundsen Sea compared to meteoric water, mainly due to its seeding effects. However, meteoric water may play a more important role in phytoplankton community changes in the Amundsen Sea. The increase in meteoric water promotes the growth of larger phytoplankton susceptible to Fe deficiencies by increasing the DFe content in the environment, which may lead to changes in phytoplankton communities and carbon exports in the SO.

## Figures and Tables

**Table 1:** Data on the CFR and FeUR values and their size-fractionated distributions in the Amundsen Sea.

Station	Depth (m)	CFR (mgC m^−3^ d^−1^)	Micro-CFR (mgC m^−3^ d^−1^)	Nano-CFR (mgC m^−3^ d^−1^)	Pico-CFR (mgC m^−3^ d^−1^)	FeUR (pmolFe L^−1^ d^−1^)	Micro-FeUR (pmolFe L^−1^ d^−1^)	Nano-FeUR (pmolFe L^−1^ d^−1^)	Pico-FeUR (pmolFe L^−1^ d^−1^)
AD-02	0	0.27 ± 0.03	0.07 ± 0.00	0.20 ± 0.03 *		14.59 ± 1.32	3.20 ± 1.08	11.40 ± 0.24 *	
A1-02	0	1.83 ± 0.71	1.20 ± 0.71	0.57 ± 0.00	0.06 ± 0.00	14.65 ± 1.84	3.87 ± 1.93	5.27 ± 0.09	5.51 ± 0.00
	25	1.34 ± 0.36	0.77 ± 0.20	0.30 ± 0.30	0.28 ± 0.16	17.58 ± 1.50	6.35 ± 1.39	4.17 ± 0.26	7.06 ± 0.37
A1-03	0	1.40 ± 0.01	0.29 ± 0.03	1.11 ± 0.04 *		15.47 ± 0.78	5.83 ± 3.07	9.64 ± 3.85 *	
A1-04	0	0.89 ± 0.00	0.08 ± 0.00	0.81 ± 0.00 *		18.19 ± 0.00	1.90 ± 0.00	16.29 ± 0.00 *	
	25	0.60 ± 0.00	0.11 ± 0.00	0.49 ± 0.00 *		20.29 ± 0.00	4.41 ± 0.00	15.88 ± 0.00 *	
A2-02	0	0.69 ± 0.12	0.31 ± 0.13	0.34 ± 0.01	0.04 ± 0.00	9.53 ± 2.21	2.61 ± 1.29	3.24 ± 2.86	3.68 ± 1.94
	50	0.41 ± 0.18	0.27 ±0.03	0.14 ± 0.01 *		5.55 ± 0.18	1.11 ± 0.18	1.44 ± 0.08	3.00 ± 0.08
A2-04	0	0.20 ± 0.29	nd	nd	nd	19.53 ± 0.92	5.15 ± 0.78	10.47 ± 0.61	3.91 ± 0.46
A2-08	0	0.16 ± 0.00	0.11 ± 0.00	0.04 ± 0.00	0.02 ± 0.00	1.68 ± 0.00	0.55 ± 0.00	0.32 ± 0.00	0.81 ± 0.00
	40	0.34 ± 0.00	0.09 ± 0.00	0.18 ± 0.00	0.06 ± 0.00	4.58 ± 0.00	1.99 ± 0.00	1.26 ± 0.00	1.32 ± 0.00
A2-09	0	0.35 ± 0.03	0.26 ± 0.02	0.05 ± 0.01	0.05 ± 0.00	38.19 ± 0.00	22.83 ± 0.00	9.76 ± 0.00	5.60 ± 0.00
A2-11	0	0.74 ± 0.06	0.56 ± 0.07	0.07 ± 0.00	0.11 ± 0.01	21.88 ± 3.23	12.17 ± 2.25	1.99 ± 1.21	7.73 ± 0.23
A2-15	0	0.82 ± 0.00	0.13 ± 0.00	0.15 ± 0.00	0.55 ±0.00	1.66 ± 0.63	1.66 ± 0.63	nd	nd
	25	0.29 ± 0.00	0.00 ± 0.00	0.08 ± 0.00	0.20 ± 0.00	5.63 ± 3.25	0.52 ± 0.26	2.87 ± 1.89	2.23 ± 1.10
A2-17	0	0.36 ± 0.04	0.05 ± 0.00	0.10 ± 0.00	0.21 ± 0.04	9.19 ± 0.79	1.72 ± 0.72	2.72 ± 1.00	4.76 ± 1.08
	75	0.29 ± 0.02	0.04 ± 0.00	0.10 ± 0.04	0.15 ± 0.06	nd	nd	nd	nd

Note: nd means no data; * represents the sum of the nano-plankton and pico-plankton.

**Table 2:** The Fe/C ratios of phytoplankton absorbing DFe and DIC in ambient seawater and with Fe addition. Note: nd represents no data.

	Depth (m)	Fe/C Ratio (μmol/mol)					
Station		Ambient			Fe Addition		
		Total	Micro-	Nano-+Pico-	Total	Micro-	Nano-+Pico-
AD-02	0	640	552	670	1783	784	2534
A1-02	0	96	39	204	1972	249	n.d.
	25	171	99	234	972	2595	679
A1-03	0	133	242	105	151	245	126
A1-04	0	245	272	243	154	66	172
	25	403	464	389	116	34	195
A2-02	0	166	101	221	76	96	215
	50	129	49	376	116	43	270
A2-04	0	297	n.d.	n.d.	322	n.d.	n.d.
A2-08	0	123	61	239	139	149	131
	40	164	253	129	262	318	236
A2-09	0	1301	1056	1982	1602	1754	1318
A2-11	0	357	262	653	662	437	391
A2-15	0	24	151	nd	14	22	nd
	25	195	19,387	212	nd	nd	nd
A2-17	0	233	384	289	nd	nd	nd

## Data Availability

The data presented in this study are available from the corresponding author upon reasonable request.

## Supplementary Materials

The following supporting information can be downloaded at: https://www.mdpi.com/article/10.3390/biology11121760/s1, Table S1: Preliminary results for the removal efficiencies of extracellular Fe by EDTA–oxalate reagent and DFe by Chelex-100 resin and the adsorbance efficiency of Fe on the filter, Table S2: The endmember salinity and *δ*^18^O values for meteoric water (MW), sea ice meltwater (SIM), and circumpolar deep water (CDW) used in the freshwater component calculation model, Figure S1: The relationship between the δ^18^O and salinity in the Amundsen Sea. The CDW end-member is shown as a circle. The upper and lower lines represent a conservative mix between the CDW and SIM or MW end-member, respectively, Figure S2: Distribution of hydrological and hydrochemical parameters in surface water: (**a**) temperature, °C; (**b**) salinity, psu; (**c**) potential density anomaly (sigma-0), kg m^−3^; (**d**) DO, μmol L^−1^; (**e**) MLD, m; (**f**) CMD, m; (**g**) DIN, μmol L^−1^; (**h**) fluorescence, μg L^−1^; (**i**) Chl-α, mg m^−3^, Figure S3: The relationship between fluorescence and Chl-α in the surface water, Figure S4: The relationships between the freshwater fraction (f_SIM+MW_) and the salinity and sigma-0 (**a**) and the MLD and CMD (**b**) in the Amundsen Sea, Figure S5: The relationships between the contributions of size-fractionated CFRs (%, left panel) and FeURs (%, right panel) and the f_MW_ (**a**,**d**), f_SIM_ (**b,e**), and f_SIM+MW_ (**c**,**f**). The solid squares and open circles represent micro-plankton and the sum of nano- and pico-plankton, respectively, Figure S6: The CFRs vary with the mixed layer depths (MLDs) and phytoplankton biomass levels (expressed as fluorescence) in the Amundsen Sea, Figure S7: The relationships between fluorescence and the f_MW_ (**a**), f_SIM_ (**b**), and f_SIM+MW_ (**c**) in the surface water, Figure S8: The relationship between the size-fractionated uptake rates (CFR (**a**) and FeUR (**c**)), their corresponding contributions (**b**, **d**), and the MLD. The solid square and open circle represent the micro-plankton and the sum of the nano- and pico-plankton, respectively.
