# Supplementary material for: Carbon and Iron Uptake by Phytoplankton in the Amundsen Sea, Antarctica
Source: Biology (Basel). 2022 Dec 4;11(12):1760. doi: 10.3390/biology11121760 (PMC9775254; doi:10.3390/biology11121760)

Supplementary Material

Carbon and Iron Uptake by Phytoplankton in the Amundsen Sea, Antarctica

Bo Wang [1,2], Lingfang Fan [1], Minfang Zheng [1], Yusheng Qiu [1] and Min Chen [1,*]

[1] College of Ocean and Earth Sciences, Xiamen University, Xiamen 361102, China
[2] College of Safety and Environmental Engineering, Shandong University of Science and Technology, Qingdao 266590, China
* Correspondence: mchen@xmu.edu.cn

Contents of this file

Table S1 to S2

Figure S1 to S8

Table S1. Preliminary results for the removal efficiencies of extracellular Fe by EDTA–oxalate reagent and the DFe by Chelex-100 resin and the adsorbance efficiency of Fe on the filter.

Table S2. The endmember salinity and $\delta^{18}O$ values for meteoric water (MW), sea ice meltwater (SIM), and circumpolar deep water (CDW) used in the freshwater component calculation model.

Figure S1. The relationship between the $\delta^{18}O$ and salinity in the Amundsen Sea. The CDW end-member is shown as a circle. The upper and lower lines represent a conservative mix between the CDW and SIM or MW end-member, respectively.

Figure S2. Distribution of hydrological and hydrochemical parameters in surface water; (a) temperature, °C; (b) salinity, psu; (c) potential density anomaly (sigma-0), kg m^{-3}; (d) DO, μmol L^{-1}; (e) MLD, m; (f) CMD, m; (g) DIN, μmol L^{-1}; (h) fluorescence, μg L^{-1}; (i) Chl-*α*, mg m^{-3}.

Figure S3. The relationships between fluorescence and Chl-*α* in the surface water.

Figure S4. The relationships between the freshwater fraction (f_{SIM+MW}) and the salinity and sigma-0 (a) and MLD and CMD (b) in the Amundsen Sea.

Figure S5. The relationships between the contributions of size-fractionated CFRs (%, left panel) and FeURs (%, right panel) and the f_{MW} (a, d), f_{SIM} (b, e), and f_{SIM+MW} (c, f). The solid and open circles represent micro-plankton and the sum of nano- and pico-plankton, respectively.

Figure S6. The CFRs varies with the mixed layer depths (MLDs) and phytoplankton biomass levels (expressed as fluorescence) in the Amundsen Sea.

Figure S7. The relationships between fluorescence and the f_{MW} (a), f_{SIM} (b), and f_{SIM+MW} (c) in the surface water.

Figure S8. The relationship between the size-fractionated uptake rates (CFR (a) and FeUR (c)), their corresponding contributions (b and d) and the MLD. The solid and open circle represent the micro-plankton and the sum of the nano-

and pico-plankton, respectively.

Table S1. Preliminary results for the removal efficiencies of extracellular Fe by EDTA–oxalate reagent and the DFe by Chelex-100 resin and the adsorbance efficiency of Fe on the filter.

Preliminary tests		Efficiency
Removal efficiency	Extracellular Fe by EDTA-oxalate reagent	94%
	DFe by Chelex-100 resin	(95.3±0.6) %
Adsorb efficiency	Fe on the filter	2%-4%

Table S2. The endmember salinity and $\delta^{18}O$ values for meteoric water (MW), sea ice meltwater (SIM), and circumpolar deep water (CDW) used in the freshwater component calculation model.

Parameter	MW endmember	SIM endmember	CDW endmember
S	0	7	34.78
$\delta^{18}O$ (‰)	-25.0	2.1	0.1

Figure S1. The relationship between the δ18O and salinity in the Amundsen Sea. The CDW end-member is shown as a circle. The upper and lower lines represent a conservative mix between the CDW and SIM or MW end-member, respectively.

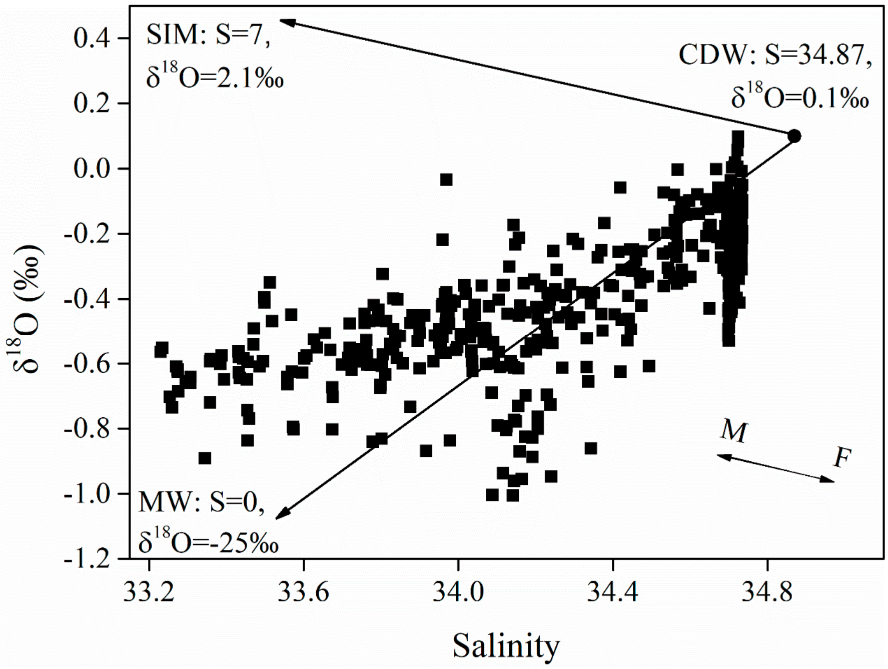

Figure S2. Distribution of hydrological and hydrochemical parameters in surface water; (a) temperature, °C; (b) salinity, psu; (c) potential density anomaly (sigma-0), kg m^{-3}; (d) DO, μmol L^{-1}; (e) MLD, m; (f) CMD, m; (g) DIN, μmol L^{-1}; (h) fluorescence, μg L^{-1}; (i) Chl-α, mg m^{-3}.

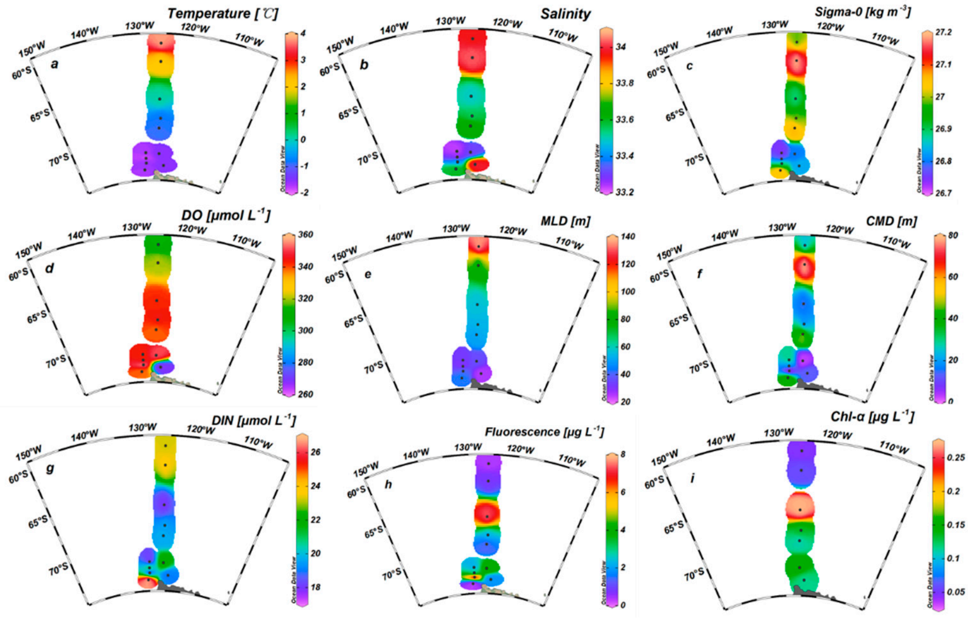

Figure S3. The relationships between fluorescence and Chl-α in the surface water.

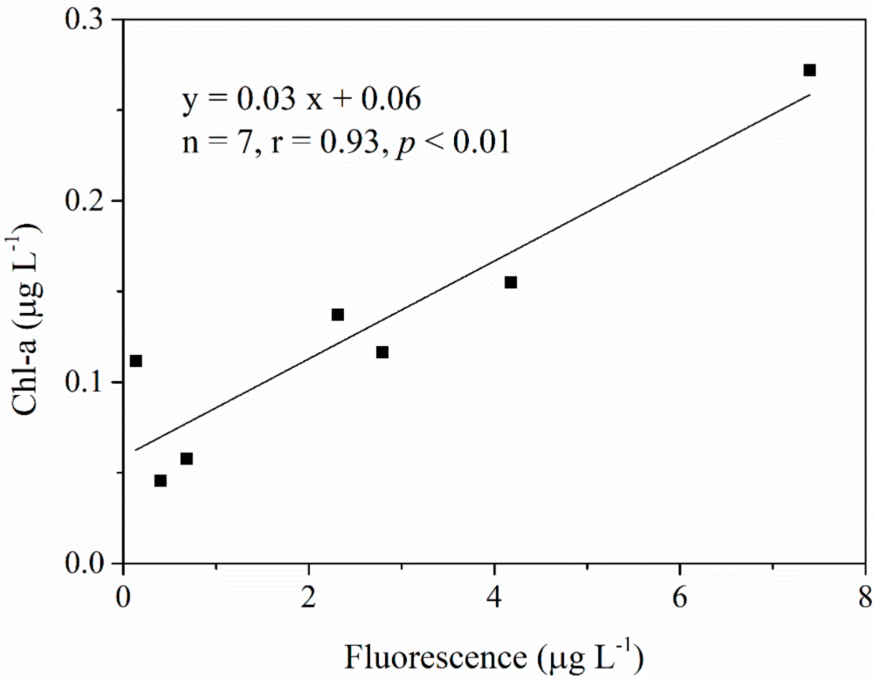

Figure S4. The relationships between the freshwater fraction (f_{SIM+MW}) and the salinity

and sigma-0 (a) and MLD and CMD (b) in the Amundsen Sea.

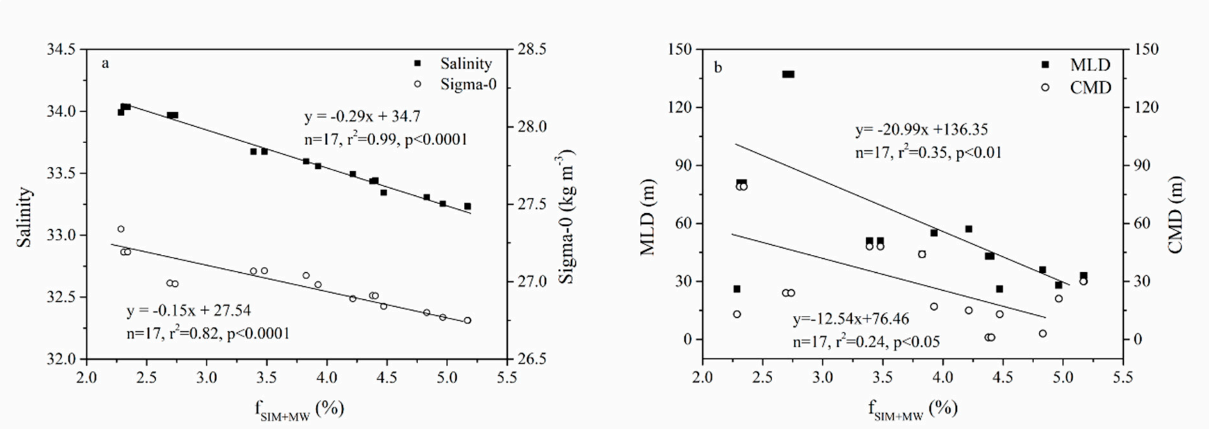

Figure S5. The relationships between the contributions of size-fractionated CFRs (%, left panel) and FeURs (%, right panel) and the f_{MW} (a, d), f_{SIM} (b, e), and f_{SIM+MW} (c, f). The solid and open circles represent micro-plankton and the sum of nano- and pico-plankton, respectively.

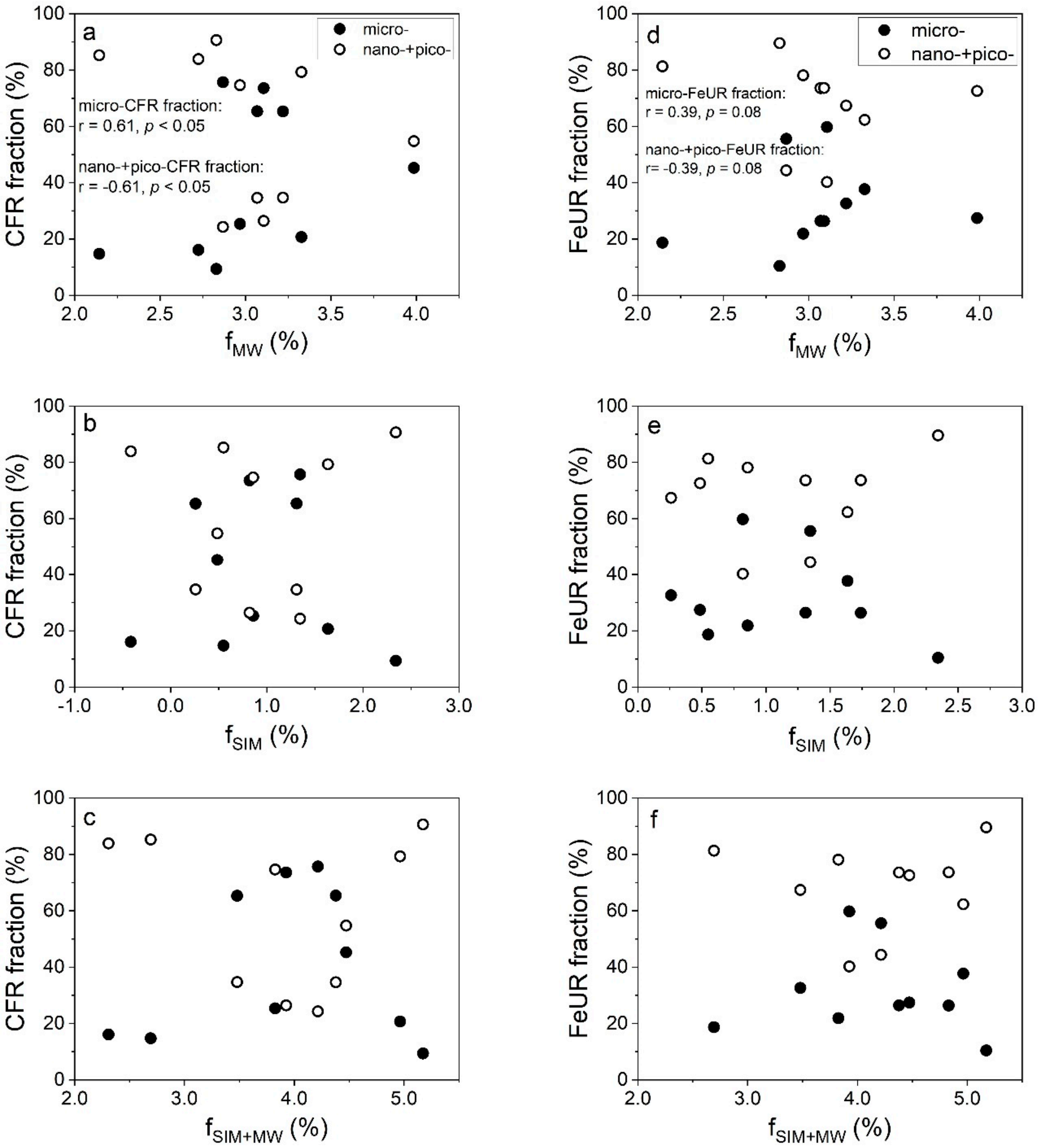

Figure S6. The CFRs varies with the mixed layer depths (MLDs) and phytoplankton biomass levels (expressed as fluorescence) in the Amundsen Sea.

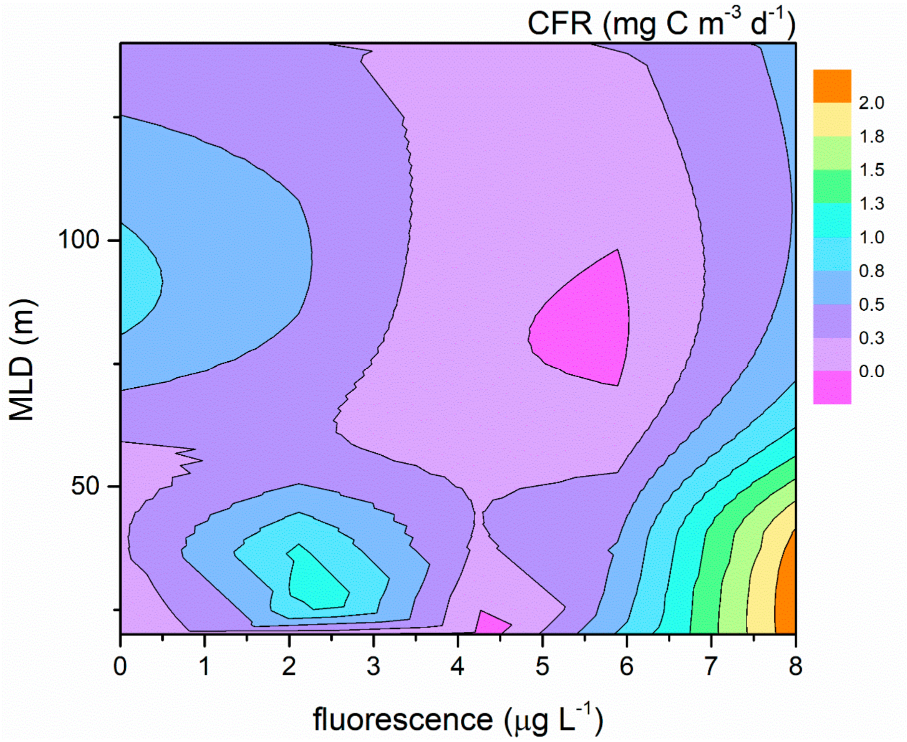

Figure S7. The relationships between fluorescence and the f_{MW} (a), f_{SIM} (b), and f_{SIM+MW} (c) in the surface water.

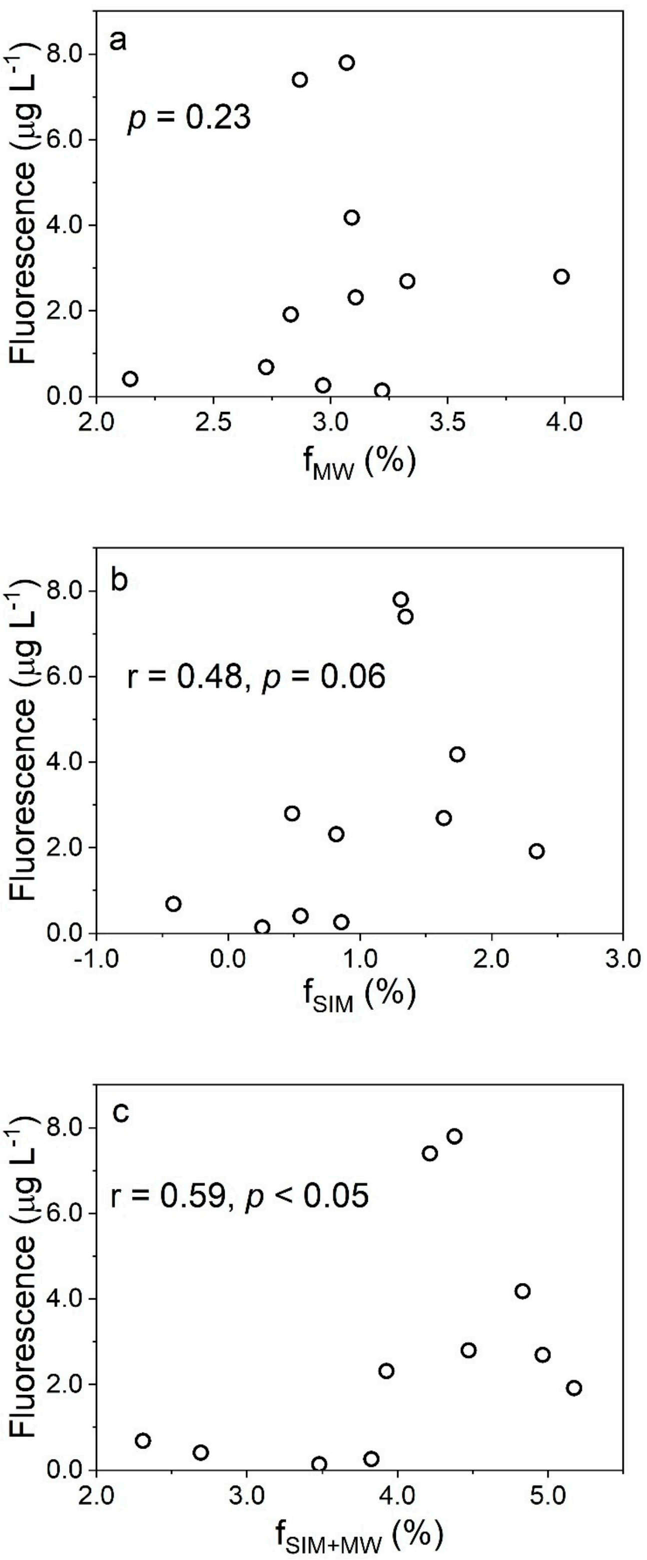

Figure S8. The relationship between the size-fractionated uptake rates (CFR (a) and FeUR (c)), their corresponding contributions (b and d) and the MLD. The solid and open circle represent the micro-plankton and the sum of the nano- and pico-plankton, respectively.

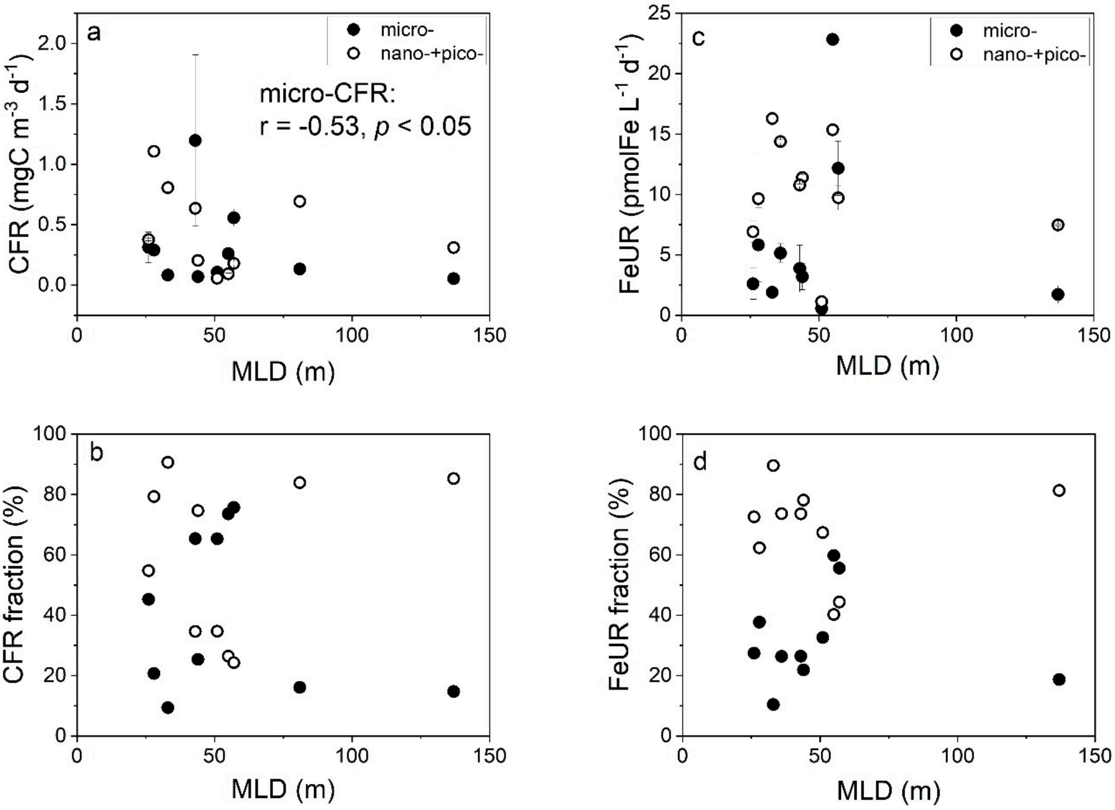

Supplement: Supplementary file 1 [file biology-11-01760-s001.zip › biology-2023647-supplementary.pdf]
